# Pediatric kidney transplantation in Europe, a clinical snapshot pilot

**DOI:** 10.3389/fped.2024.1432027

**Published:** 2024-10-24

**Authors:** Loes Oomen, Charlotte M. H. H. T. Bootsma-Robroeks, Antonia H. M. Bouts, Mar Carbonell Pradas, Romy Gander, Katrin Kienzl-Wagner, Paul König, Pedro Lopez Pereira, Olivier Dunand, Sara M. F. S. Mosca, Michal Pac, Ludmila Podracka, Agnieszka A. Prytula, Maria Sangermano, Renata Vitkevic, Jakub Zieg, Loes F. M. van der Zanden, Wout F. J. Feitz, Liesbeth L. de Wall

**Affiliations:** ^1^ Division of Pediatric Urology, Department of Urology, Radboudumc Amalia Children’s Hospital, Nijmegen, Netherlands; ^2^ Department of Pediatric Nephrology, Radboudumc Amalia Children’s Hospital, Nijmegen, Netherlands; ^3^ Department of Pediatrics, Pediatric Nephrology, Beatrix Children’s Hospital, University of Groningen, University Medical Centre Groningen, Groningen, Netherlands; ^4^ Department of Pediatric Nephrology, Emma Children’s Hospital, Amsterdam University Medical Center, Amsterdam, Netherlands; ^5^ Department of Pediatric Surgery, Hospital Sant Joan de Déu, Universitat de Barcelona, Barcelona, Spain; ^6^ Pediatric Urology and Renal Transplant Unit, Department of Pediatric Surgery, University Hospital Vall d´Hebron Barcelona, Barcelona, Spain; ^7^ Department of Visceral, Transplant and Thoracic Surgery, Medical University of Innsbruck, Innsbruck, Austria; ^8^ Department of Urology and Pediatric Urology, Universitätsklinikum Erlangen, Friedrich-Alexander-Universität Erlangen-Nürnberg, Erlangen, Germany; ^9^ Department of Pediatric Urology, University Hospital La Paz, Madrid, Spain; ^10^ Department of Pediatric Nephrology, University Hospital of Réunion, La Réunion, France; ^11^ Department of Pediatric Nephrology, Centro Materno Infantil do Norte, Centro Hospitalar Universitário de Santo António, Porto, Portugal; ^12^ Department of Nephrology, Kidney Transplantation and Hypertension, Children’s Memorial Health Institute, Warsaw, Poland; ^13^ 1st Dept Pediatric Children’s Hospital, Comenius University, Bratislava, Slovakia; ^14^ Department of Paediatric Nephrology and Rheumatology, Ghent University Hospital, ERKNet Centre, Ghent, Belgium; ^15^ Pediatric Nephrology, Dialysis and Transplant Unit, Department of Women’s and Children’s Health, Padua University Hospital, Padua, Italy; ^16^ Department of Pediatrics, Vilnius University Hospital Santaros Klinikos, Vilnius, Lithuania; ^17^ Department of Pediatrics, Second Faculty of Medicine, Charles University and Motol University Hospital, Prague, Czechia; ^18^ IQ Health Science Department, Radboudumc, Nijmegen, Netherlands

**Keywords:** pediatric kidney transplantation, clinical practice snapshot, Europe, donor type, graft survival, registries

## Abstract

**Background:**

Pediatric kidney transplantations are rarely performed, and there is limited knowledge about the diversity in current clinical practices across Europe. This study aims to explore the utility of clinical snapshot studies in identifying these disparities, establishing a foundation for future snapshot studies and standardization efforts.

**Methods:**

A pilot clinical snapshot study was conducted, with invitations extended to all 109 pediatric kidney transplant centres in Europe. Each participating centre provided pre-, peri-, and postoperative data concerning their most recent thirty transplantations. The primary outcomes encompassed the evaluation of disparities in donor-recipient selection, surgical techniques, post-operative drainage procedures, and immunosuppressive therapy protocols. Secondary outcomes involved the analysis of rejection rates, incidence of infections, and graft survival.

**Results:**

The study involved 439 patients from fifteen centres (14%) in twelve countries, with varying transplant volumes (range 1–29 transplantations per year) and follow-up periods. Significant differences were found among centres in terms of donor types, cold and warm ischemia time, pre-emptive transplant rates, and kidney transplant drainage methods. The rate of living donors varied between 3% and 90% and the median duration of cold ischemia ranged was 770 min after deceased donation and 147 min after living donation. Basiliximab was the dominant induction therapy, yet steroid withdrawal varied widely. Infection, rejection, and graft survival rates also varied significantly between centres.

**Conclusion:**

This study revealed substantial variation in clinical practices among European centres performing pediatric kidney transplantations. These findings could serve as a stimulus for international dialogue and collaboration.

## 1 Introduction

Pediatric kidney transplantations (PKT) remain a rarity, with only 500–600 performed in Europe annually, compared to over 21,000 procedures in adults (1, 2). Previously, we identified 109 centres within the European Union that perform PKT revealing significant variations in the level of centralization across countries (3). This raises questions about the divergence in clinical practices throughout Europe. Considering the improvements in immunosuppressive strategies, surgical techniques, and utilization of living (unrelated) donors (LD) in recent decades, there might be a considerable variation in daily practices among European centres and countries. Despite the presence of national PKT protocols, international guidelines are primarily designed for adult transplants with no specific European guideline for PKT (4, 5).

For enhanced coordination and care evaluation, gaining better insights into current practices is of importance. This information might allow comparison of clinical management among expert centres, replication of best practices in different countries, and improving the quality of care where possible. The low incidence of PKT poses challenges for traditional research methods. A recent publication showed that only 39% of the PKT centres contribute their data to existing European, multi-national registries, and these registries show substantial differences in the parameters they collect (3). Besides, information on peri-operative urological factors is lacking in all of these registries. Therefore, this multi-centre retrospective cohort study aims to explore variations in clinical practices across Europe marking the initial step toward potential standardization efforts.

## 2 Methods

### 2.1 Study design

This clinical snapshot pilot is an international retrospective cohort study. Snapshot studies allow researchers to efficiently collaborate with numerous clinicians, and to swiftly collect data from a diverse range of patients within a short timeframe. In this pilot, the use of snapshot studies to explore clinical variety is examined.

### 2.2 Study process

An electronic Case Report Form (eCRF) was developed to obtain information about the clinical practice of PKT. The variables were based on existing literature on prognostic factors influencing transplantation outcomes and the expertise and consensus of five expert clinicians. The eCRF covered six primary topics with a total of thirty-three questions. These topics included Characteristics (recipient, donor and transplantation), Surgical parameters, Graft Function, Immunosuppression, Infection, and Rejection (Supplementary Material 1).

Between March 2022 and November 2022, all 109 PKT centres across Europe were invited to participate, with a reminder sent after 1 month to non-responsive centres. To ensure anonymity, participating centres were identified by codes (e.g., C1, C2).

Data was collected from November 2022 to July 2023. Each participating centre contributed pseudonymized retrospective data for the last thirty consecutive patients who underwent PKT (The number of thirty patients was chosen to ensure a balance between data quantity, quality, and feasibility. While the primary focus was pediatric patients under nineteen, some centres extended the age range to include those up to 20 years.

Patients who received multiorgan transplants or had less than 1 month of observation were excluded due to differing clinical management and limited information. Only patients with a follow-up duration of at least 6 months were included in the analysis of follow-up events.

Data underwent rigorous validation through initial analysis by the primary researcher and cross-verification by individual centres to ensure reliability. Pseudonymized research data were securely entered into CastorEDC, a password-protected, cloud-based clinical data platform, with specific roles assigned (e.g., study coordinator, investigator). Data management and monitoring took place within CastorEDC.

The Committee for Human Research from Radboudumc waived the requirement for informed consent (file number: 2022-16138), respecting patient privacy and confidentiality. The study report adhered to the STROBE guidelines, with the comprehensive STROBE checklist available in Supplementary Material 3.

### 2.3 Definitions

To enhance the readability of the manuscript and facilitate data collection, causes of kidney failure were divided into two categories: urological conditions (vesico-ureteral reflux, neurogenic bladder, or posterior urethral valves) and nephrological conditions (all other cases). Immunosuppressive therapy was split into two categories as well: induction therapy (specific drugs given before surgery) and maintenance therapy (immunosuppressive medication prescribed at discharge after transplantation). In small children where extraperitoneal placement is not feasible, the graft may be placed intra-abdominally, defined as intraperitoneal kidney transplant with anastomosis within the abdominal cavity (6). Follow-up duration was calculated as the time between the last visit and the transplantation date.

Graft function was assessed by creatinine clearance, calculated using the Schwartz formula to estimate glomerular filtration rate (eGFR) with a maximum of 90 (7). This approach was chosen due to the widespread availability of its parameters, in contrast to more sophisticated methods for measuring renal function (8). Graft loss was defined as the start of dialysis or re-transplantation, while patient deaths with functioning grafts were considered as graft survival. Rejection was defined as a biopsy-proven acute rejection using the Banff classification (9).

Urinary tract infection (UTI) was defined as urine culture demonstrating the presence of >50,000 colony forming units/ml of a single pathogen in combination with clinical symptoms. Cytomegalovirus (CMV) and Epstein-Barr virus (EBV) infections were defined as the presence of CMV/EBV viremia or DNAemia at any point during follow-up (10). BK-virus (BKV) replication was quantified using real-time quantitative PCR assay using BKV specific primers. Infection and rejection rates were calculated per year of follow-up, while graft deterioration was measured as the change in eGFR from discharge to the last known value, divided by follow-up time.

### 2.4 Statistical analysis

Baseline characteristics of the study cohort were presented as numbers/percentages, median/interquartile ranges (IQR)/total ranges for skewed data or mean/standard deviation (SD) for normally distributed data. Missing data were handled through pairwise deletion.

To examine potential variations in patient characteristics, protocols, and outcomes among centres, chi-square tests, one-way ANOVA, and logistic regression analyses were used, all adjusted for follow-up duration. Logistic regression parameters were limited to a maximum of 10% relative to total events.

Graft survival curves were constructed using the Kaplan-Meier method and assessed with the log-rank test to identify significant differences in graft survival among groups. Statistical significance was defined at *p* < 0.05. Data analysis was performed using SPSS Statistics 25.0.

## 3 Results

**Figure 1:**
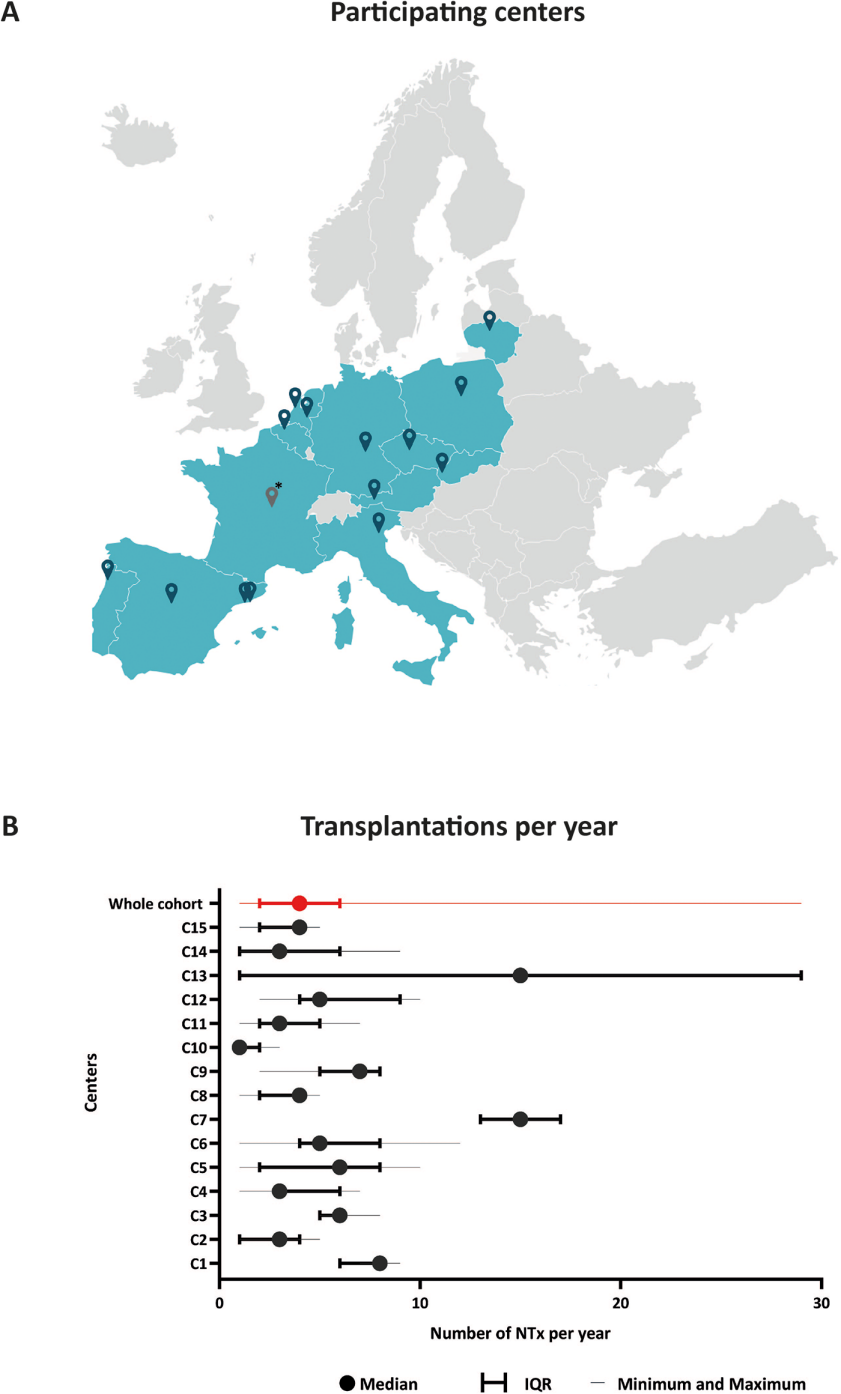
Overview of participating centers. **(A)** Geographical distribution of participating centers within Europe. **(B)** Number of pediatric kidney transplantations performed per year (*n* = 439). * Located in La Réunion, a French island not shown on this map. IQR, interquartile range; NTx, kidney transplantation.

Of 109 invited centres, 71 did not respond and 23 centres provided reasons for their non-participation such as lack of time/resources (*N* = 10, 43%) and local legislation (*N* = 5, 22%). Ultimately, fifteen centres (14%) in twelve countries* contributed patient data (Figure 1A) (* Austria, Belgium, Czechia, Germany, France, Italy, Lithuania, the Netherlands, Poland, Portugal, Slovakia, Spain).

Data from 439 transplantations (2011–2023) were available for analysis. Eleven patients were missing from one centre since this centre only recently started PKT. Median follow-up time was 26 months (range: 1–140 months, IQR: 10–46 months). The median annual transplantation rate was 4 (range: 1–29, IQR: 2–6), indicating varying degrees of centralization (Figure 1B).

### 3.1 Recipient/transplantation characteristics

**Figure 2:**
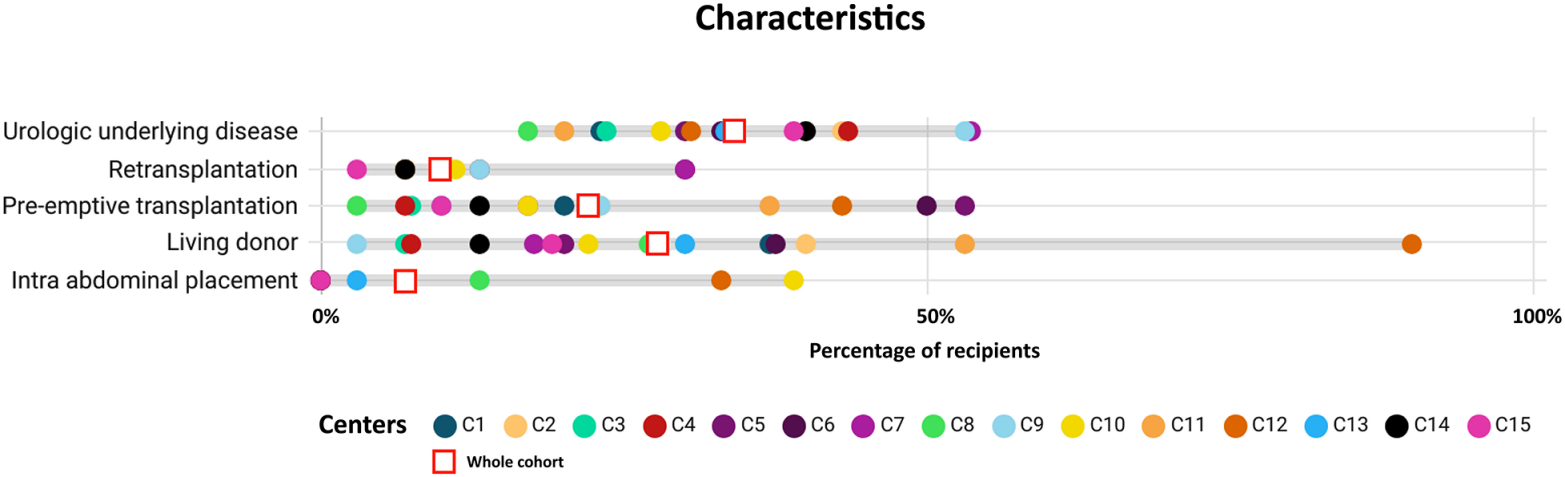
Comparison of main characteristics by centre. Percentage of recipients depicted by centre (*n* = 439). All characteristics were statistically significant (*p* < 0.05) except for underlying disease. Urologic underlying disease is defined as either vesico-ureteral reflux, neurogenic bladder, or posterior urethral valves. Intra-abdominal placement is defined as intraperitoneal placement of the kidney transplant with anastomosis within the abdominal cavity.

Significant variations were observed in patient and transplantation characteristics across participating centers (Figure 2, Supplementary Table 1). All characteristics showed statistical significance except for the underlying disease causing renal failure (*p* = 0.16).

The type of donor also exhibited considerable disparities, with the majority of transplantations being performed after deceased donation (DD), 14 (3%) after circulatory death, 287 (65%) after brainstem death. Only one center showed a significant difference in recipient age between LD and DD transplants (Supplementary Material 2, Supplementary Figure 1). Furthermore, all centers included in the study performed transplants in recipients under the age of five, with five centers specifically conducting transplants in children younger than 3 years old (Supplementary Material 2, Supplementary Figure 1).

While it might be expected that LD would be more commonly used in pre-emptive transplantations, as seen in C3 and C4, this pattern was not consistent across all centers. For example, in C5 and C9, the majority of pre-emptive transplantations were conducted with DD (Supplementary Material 2, Supplementary Figure 2).

The number of human leucocyte antigen (HLA) mismatches showed significant differences with five centres permitting up to 6 mismatches, while 3 centres had a maximum allowance of 4 mismatches.

### 3.2 Surgical parameters

#### 3.2.1 Peri-operative parameters

In 16% of the patients, the graft was placed intraperitoneal (intraabdominal placement). The age associated with intra-abdominal placement differed across centers. Three centers exclusively reserved this approach for recipients under 5 years old, while four centers employed it for recipients of all ages.

**Figure 3:**
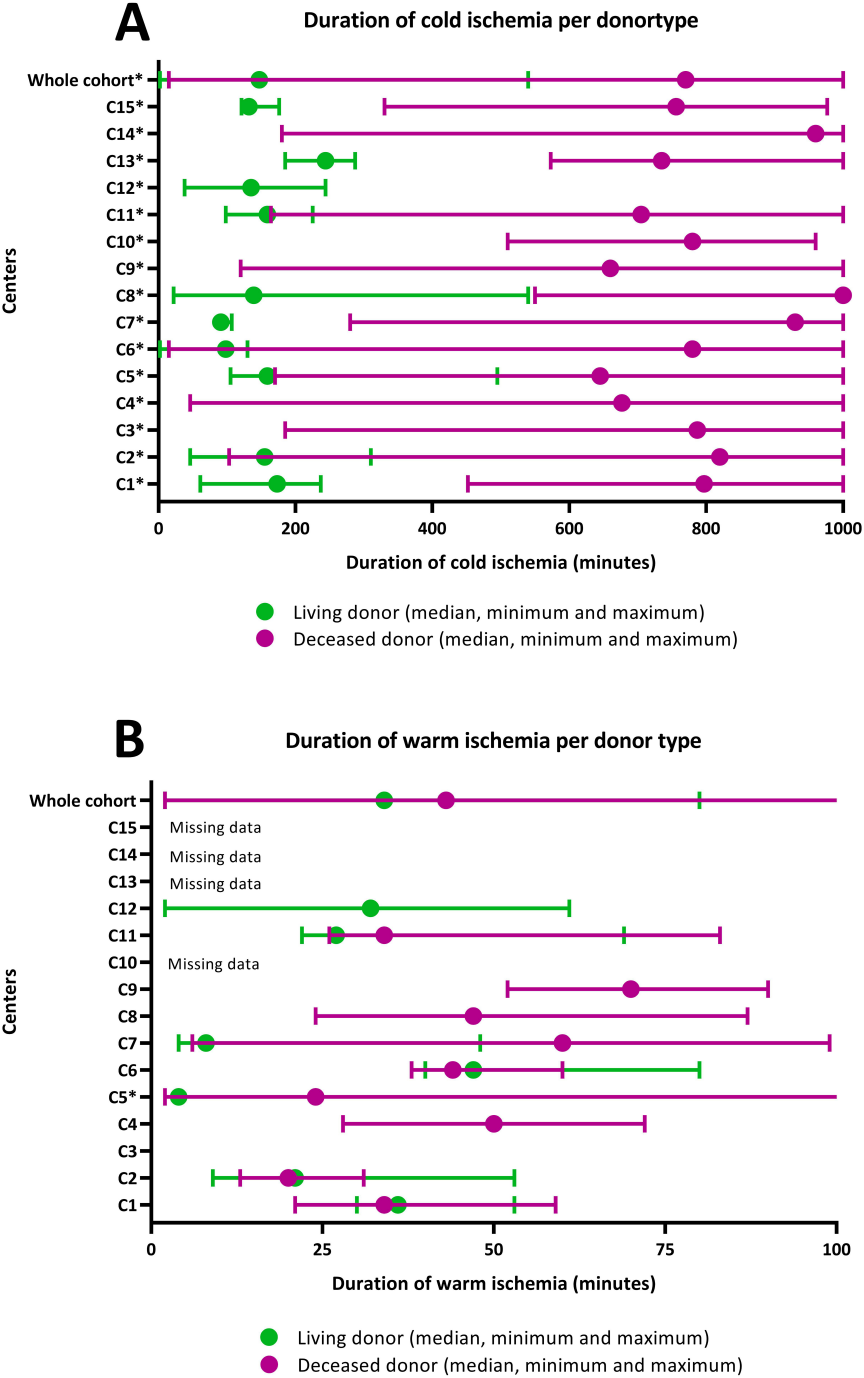
Duration of ischemia by centre. **(A)** Minutes of cold ischemia time per donor type, median with minimum and maximum (*n* = 384). **(B)** Minutes of warm ischemia time per donor type, median with minimum and maximum (*n* = 267). The default maximum cold ischemia time was set at 1,000 min. When *n* < 3 data were not shown. *Indicates a significant difference between donor types.

Furthermore, the ischemia duration showed wide variability, even when adjusted for donor type (*p* < 0.05) (Figure 3A,B). The median duration of cold ischemia time (CIT) was 770 min (range 15–1,000, IQR 600–968) for DD and 147 min (range 2–540, IQR 106–192) for LD. No differences in duration of warm ischemia (WIT) were found between LD and DD.

#### 3.2.2 Post-operative parameters

**Figure 4:**
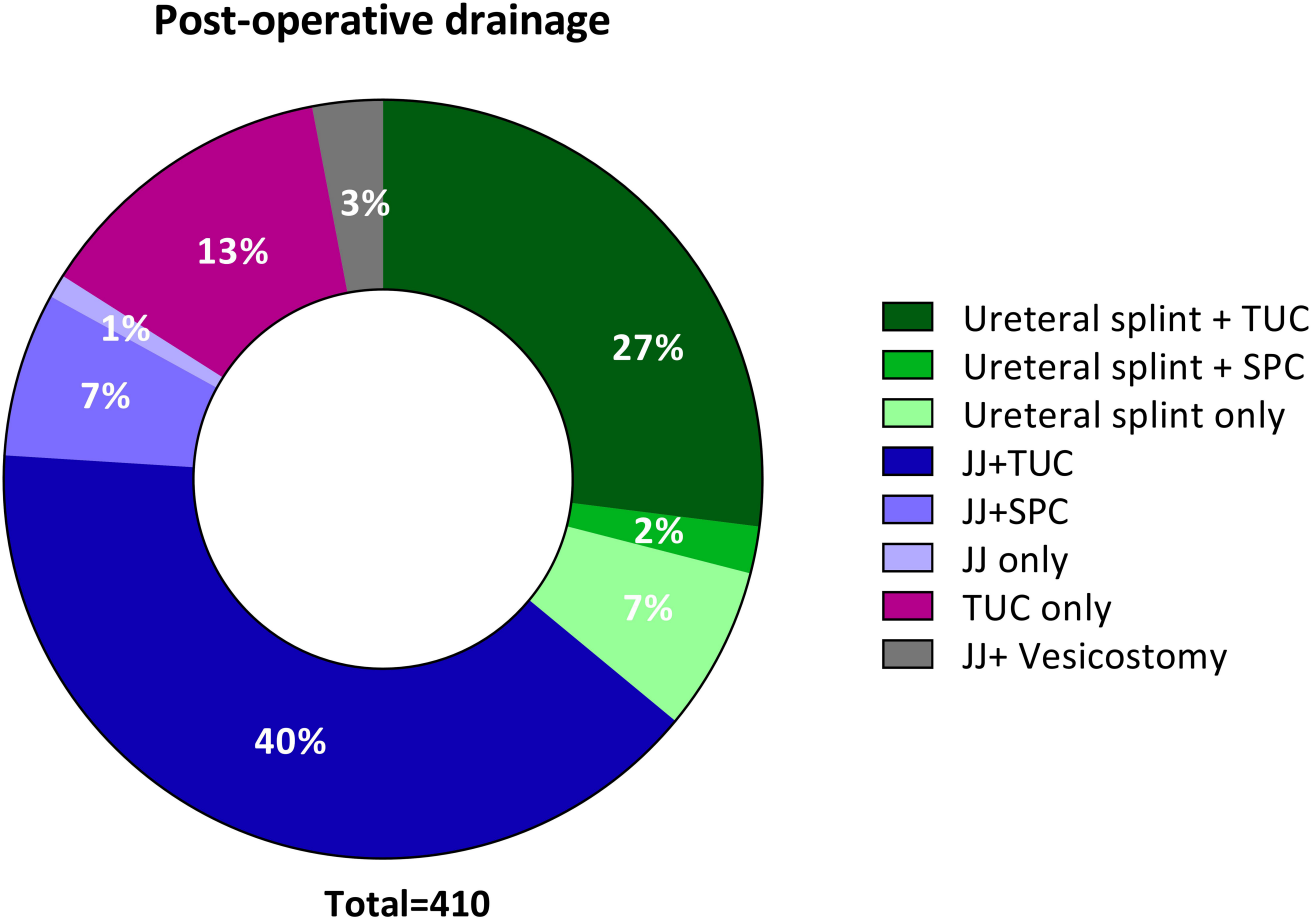
Post-operative kidney and bladder drainage (*n* = 410). JJ, Double J stent; SPC, suprapubic catheter; TUC, transurethral catheter.

Significant variations were observed in post-operative drainage protocols among the centres (*p* < 0.01). Figure 4 illustrates that the prevailing approach combined a transurethral catheter with either a double J stent (JJ) or a ureteral splint. Notably, eleven patients had a vesicostomy at the time of transplant, with the majority (*N* = 10, 91%) having a urological cause of kidney failure.

The median bladder drainage duration was 8 days [IQR 5–11] (*n* = 169). For those using a ureteral splint, the median drainage duration was 7 days [IQR 6–10] (*n* = 155), while for JJ drainage, it was 38 days [IQR 21–51] (*n* = 199). However, a substantial amount of data regarding bladder drainage duration was missing (*N* = 190) as it could not be retrieved from patient files. The method of drainage did not influence the outcome of transplantation in terms of urinary tract infections (*p* = 0.08) or graft failure (*p* = 0.79).

### 3.3 Immunosuppressive regimens

**Figure 5:**
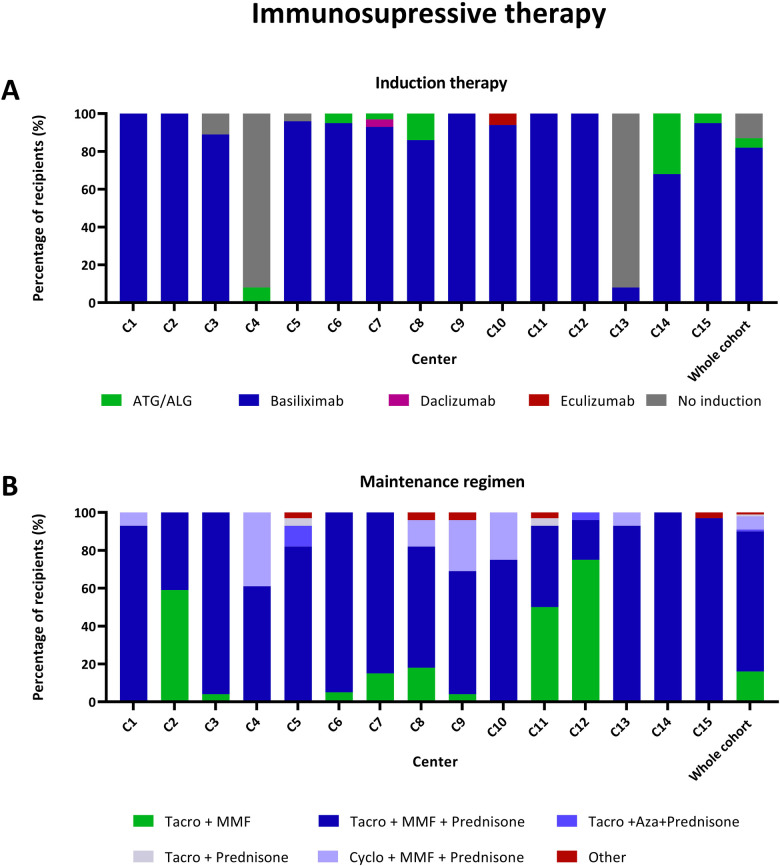
Immunosuppressive regimens in recipients that received their first transplantation (*n* = 384). **(A)** Immunosuppressive induction therapy by centre. **(B)** Immunosuppressive maintenance regimen prescribed at discharge by centre. Other: Tacrolimus monotherapy (*n* = 3), MMF + Prednisone (*n* = 2). ATG/ALG, Antithymocyte globulin/Antilymphocyte globulin; Aza, Azathioprine; MMF, Mycophenolate mofetil; Tacro, Tacrolimus.

Basiliximab served as the primary induction therapy agent (Figure 5A), followed by Antithymocyte globulin or Antilymphocyte globulin (ATG/ALG). Notably, C4 and C13 refrained from using any immunosuppressive medication prior to transplantation in most of their patients.

Upon discharge, the prevailing immunosuppressive regimen for most recipients involved a combination of a calcineurin inhibitor, mycophenolate mofetil, and prednisone (Figure 5B). However, in four centres, a considerable number of recipients followed a steroid-sparing regimen, where prednisone was not prescribed upon discharge.

### 3.4 Infections

CMV prophylaxis usage varied among centres, ranging from 27% to 97% (Supplementary Material 2, Supplementary Figure 3). A significant correlation was found between CMV prophylaxis and the CMV donor-recipient status, with most centres employing prophylaxis more often when the donor-recipient CMV status posed a higher risk of subsequent infections (D + R−). CMV prophylaxis was not associated with the number of CMV infections per year (*p* = 0.41).

Differences in CMV and BKV infections were observed between centres (*p* < 0.01 and *p* = 0.02, respectively) after adjusting for the duration of follow-up (Supplementary Material 2, Supplementary Figure 4). There was no difference in EBV infections (*p* = 0.08). The number of UTI differed across centres (*p* = 0.01), however no information on antibiotic prophylaxis was available.

The rate of EBV infections was related to the use of prednisone, even when corrected for the EBV status of the donor and recipient prior to transplantation (Supplementary Material 2, Supplementary Table 2). The use of prednisone at discharge significantly increased the risk of EBV infection during the follow-up period.

### 3.5 Graft outcome

**Figure 6:**
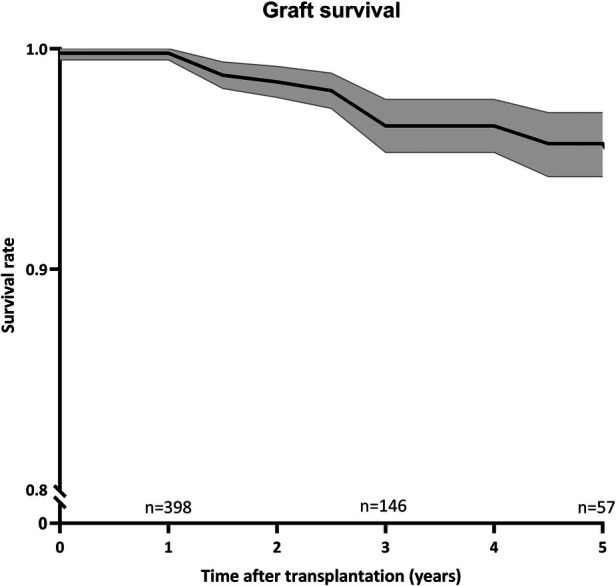
Graft survival over time. Kaplan-Meier curve for graft survival with 95% confidence interval (*n* = 439). Number of remaining cases were indicated every 2 years.

Figure 6 illustrates graft survival with a considerable 95% confidence interval after 5 years, suggesting variability in graft outcomes. Graft survival varied between centres (*p* = 0.02), as did the incidence of biopsy-proven rejection (*p* < 0.01) (Supplementary Material 2, Supplementary Figure 4). Rejection episodes occurred in 12% of recipients within the first year post-transplant, with rates varying across centres (ranging from 3% to 43%, *p* < 0.01). Despite these findings, the sample size in this study was insufficient to identify significant risk factors for graft survival.

## 4 Discussion

This pilot snapshot study on pediatric kidney transplantation practices revealed substantial variations in clinical care across European centers. These variances encompass donor-recipient selection, transplantation volume, immunosuppressive regimens, kidney and bladder drainage, as well as differences in transplantation outcomes across centers. Additionally, the annual transplantation rates vary among centers. This prompts further inquiry into the ideal proficiency, expertise threshold and potential merits of further centralizing. Contento et al. reported higher graft survival in high-volume centers (>8 per year) compared to low-volume centers (<4 per year) (11). While centralizing PKT care may enhance outcomes by ensuring collective expertise of a multidisciplinary team, these advantages should be outweighted against disadvantages like geographical, logistical, and financial factors within countries (2, 12).

### 4.1 Pre-operative parameters

The main modifiable pre-transplant factor is donor selection, favoring LD over DD when feasible (13, 14). LD transplantations ranged between 3% and 90%, suggesting differences in protocols and allocation systems throughout European centers (2). Prioritizing pediatric patients on waiting lists may result in a higher number of young DD being allocated to these patients (1). The Council of Europe recommends the use of living (un)related donors as an opportunity to expand the number of potential donors, despite common hesitations (15).

Variations in allocation may partly explain differences in preemptive transplantations. Despite the advantages of pre-emptive transplantation, most patients underwent dialysis before their transplant. Differences could potentially be influenced by factors like varying acceptance of HLA mismatches (2, 16, 17). To choose pre-emptive transplantation with a suboptimal donor (e.g., DD, full mismatch) or to wait for an ideal donor (LD, full match) while on dialysis poses a complex dilemma (if feasible). Debates and establishing evidence-based international guidelines could be useful for improving long-term outcomes. Unfortunately, this study did not collect detailed donor information, such as age, comorbidities, or details about the composition of the surgical team. However, these factors would be valuable areas of focus for future research.

### 4.2 Peri-operative parameters

Peri-operative factors encompass a range of elements, including the surgical procedure itself, healthcare providers involved, and patient characteristics, all of which collectively influence outcomes. CIT and WIT are established determinants of transplantation success (18, 19). Variations in ischemia times persist between centers, influenced by organ procurement, transportation processes, and geographical distances across Europe (2).

While CIT primarily reflects allocation policies and travel distances, necessitating continuous international attention, variations in WIT may indicate modifiable local factors, such as surgical practices. Beyond patient-specific factors (vasculature, body mass), differences in surgical skills, techniques (e.g., intra- or extraperitoneal placement), and the surgeons’ specialties (transplant, vascular, urology, pediatric) might impact outcomes. Lack of more centralized care may lead to disparities in surgeons’ experience and expertise (18). Donor nephrectomy method (laparoscopic vs. open) could also impact WIT, though this study did not collect this data.

The choice of post-operative drainage method and duration remains an ongoing debate, balancing prevention of post-operative complications against infection risks (20, 21).

### 4.3 Post-operative factors

Despite the introduction of steroid-sparing regimens in 2012, many of the contributing centres still rely on prednisone for maintenance therapy after a first transplantation (22, 23). Uncertainty about the metabolic benefits and long-term safety of corticosteroid withdrawal were reported earlier, despite previous research demonstrating its safety in selected patients (23, 24). In this study, the use of prednisone as maintanance therapy was associated with EBV infection, although bias might exist. In children, steroid withdrawal should be seriously considered due to the potential impact of steroids on growth and development. Tönshoff et al. advocate for everolimus use to enable both steroid withdrawal and reduced exposure to calcineurin inhibitors, though none of the recipients in this study received everolimus at discharge after transplantation (25). Future research should focus on long-term effects of steroid withdrawal and identify obstacles to discontinuing prednisone. Ongoing advancements in immunosuppressive medications, including new drugs like belatacept could optimise future care.

### 4.4 Outcome

Due to the limited number of participating centers, patients, and the relatively short follow-up time, the study refrains from drawing definitive conclusions on transplantation outcomes. However, some variability in reported outcomes was observed. Generally, rejection rates in the first year were consistent with existing literature (26). However, significant discrepancies among centers, including one reporting a 43% rejection rate, raise questions about the uniformity of definitions. This variation may also relate to the use of protocol biopsies, a topic under ongoing debate. In-depth research should focus on differences and the impact and cost-effectiveness of protocol biopsies.

The 5-year graft survival rate, consistent with other research findings, was notably high at 92%, with few retransplantations performed (13, 26, 27). Long-term outcomes may vary between centers, and although the relation between clinical practice and graft survival is essential for future decision-making this falls beyond the scope of this study.

### 4.5 Future perspectives

The extensive variability in this study may stem from differences in work processes, education, individual habits, and notably, the absence of standardized PKT guidelines. Likewise, Voet et al. recently revealed a large variation in anesthesia and ICU care in pediatric kidney transplantation centers across Europe (28). The lack of international PKT guidelines may be partly attributed to local legislative disparities and variations in the roles and levels of involvement of specialists across centers. These include pediatric and adult urologists, nephrologists, transplantation surgeons, and general surgeons.

This reported diversity offers a valuable learning opportunity. Further snapshot research could help assess the impact of clinical practice diversity on transplant outcomes and assess the potential benefits of establishing best practices. Developing specific guidelines can serve as a valuable roadmap for clinicians, ensuring consistency and improve quality. The integration of evidence-based protocols with personalised medicine holds the potential to enhance the furhter optimisation of PKT care.

Addressing knowledge gaps remains crucial for evidence-based medicine. Despite the existence of multiple PKT registries in Europe, data collection is still scattered and incomplete. Further collaborations among international registries could prove valuable in aggregating new data and studying effects of various protocols on a larger scale. The establishment of European Reference Networks (ERNs) for rare and complex diseases represents a significant advancement in fostering international collaboration and, probably the harmonization of care (29, 30). Currently, three ERNs are involved in the field of pediatric kidney transplantation (PKT): ERKNet, ERN eUROGEN, and ERN TransplantChild. A collaborative effort between these networks to initiate a prospective study on variations in clinical practice would be a valuable step in the advancement of PKT care.

### 4.6 Strengths and limitations

This is a unique study in the field of PKT, providing valuable insights and a first step for further optimising PKT care. Its strengths stem from the diversity of the cohort, encompassing participants from various European countries, including those often overlooked in international analyses. Rigorous data validation processes enhance reliability of findings, and the relatively short study duration provides a snapshot of current practices.

However, a significant limitation is the restricted number of collaborating centers (14%) affecting the generalizability of the findings. This study provides only a sample from European centers rather than a comprehensive representation. However, incorporating data from more centers would likely increase the observed variability. Additionally, some definitions and measurement tools are not state of the art due to variations in diagnostics and classifications across centers. Therefore, less advanced definitions were employed to facilitate the inclusion of more patients. The substantial amount of missing and unreliable data underscores the significance of thorough documentation.

### 4.7 Conclusion

This inventory of pediatric kidney transplantation (PKT) practices in Europe revealed a wide range of clinical approaches among a sample of European centers. It serves as a stimulus for international dialogue and collaboration, emphasizing the importance of a unified effort within the transplantation community.

## Data Availability

The original contributions presented in the study are included in the article/Supplementary Material, further inquiries can be directed to the corresponding author.
