# Supplementary material for: Pediatric kidney transplantation in Europe, a clinical snapshot pilot
Source: Front Pediatr. 2024 Oct 24;12:1432027. doi: 10.3389/fped.2024.1432027 (PMC11540619; doi:10.3389/fped.2024.1432027)
Supplement: Supplementary file 2 [file Datasheet1.pdf]

Supplementary Material

1: ELECTRONIC CASE REPORT FORM

General information

1.1 Type or select from the calendar button the patient's date of birth. (YYYY)

1.2 Select the birth sex of the patient.
- Male
- Female
- Undetermined: when anatomically visible but cannot make a difference.
- Foetus (Unknown): before foetus's sex is defined and/or known.

1.3 Patient status. Select between the options:
- Alive
- Dead: please select if a patient dies after being included in the registry
- Lost in follow-up: please select if a patient is not being treated at by your HCP
- Opted-out: please select if a patient withdraws his/her Informed consent

Note: If you select "Dead", the dependent field "Date of death" will appear.

Recipient/ Transplantation characteristics

1.4 Type of underlying disease causing kidney failure
- Urological
- Nephrological

We define urological disease as obstructive uropathy such as VUR, neurogenic bladder and PUV. Other diseases can be classified as nephrological

1.5 Previous therapy: Select the option that best fits the patients history of renal replacement therapy. Pre-emptive means that no renal replacement therapy has been given before.
- Hemodialysis
- Peritoneal dialysis
- Both hemo- and peritoneal dialysis
- Pre-emptive transplantation
- Unknown

1.6 History of nephrectomy of native kidneys: indicate whether the native kidneys of the patient were removed before the transplantation.
- 0
- 1
- 2

- Unknown

1.7 Number of previous kidney transplantations received
- 0
- 1
- 2
- 3 or more
- Unknown

Surgical parameters

1.8 Date transplantation: Please fill In the date of the most recent kidney transplantation. All data entered are on the most recent transplantation. (DD-MM-YYYY)

1.9 Please select what type of donor was used for this transplantation. We define a 'living related donor' as up to third degree relatives (uncles, aunts, nephews, nieces)
- Donation after circulatory death
- Donation after brainstem death
- Living unrelated donor
- Living unrelated donor
- Unknown

1.10 Total number HLA-mismatches. Please fill In the total number of mismatches on HLA-A, HLA-B and HLA-DR (options 0-6).

1.11 HLA-DR mismatches: please fill In the total number of HLA-DR mismatches If available.
- 0
- 1
- 2
- Unknown

1.12 Placement: please Indicate where the kidney graft was placed, either intra-peritoneal (with anastomoses to aorta and vena cava) or retroperitoneal (conform the adult procedure, anastomosis to the Iliac vessels)
- Intra-peritoneal
- Retroperitoneal
- Unknown

1.13 Warm ischemia time in minutes
Warm ischemia consists of 2 periods. Please enter the sum of both ischemia periods. First warm ischemia time (ischemia in donor) is the ischemia during organ retrieval, from the time of cross clamping (or of asystole in non-heart-beating donors), until cold perfusion is commenced. Second warm ischemia time (ischemia in recipient) is the period which begins at the time of removal of the kidney from storage ice and ends with the initiation of graft reperfusion.

1.14 Cold ischemia time in minutes
This is the period after the cessation of circulation (when perfusion begins with preservation solutions) until the beginning of the vascular anastomosis in the recipient.

1.15 Post-operative drainage. Please indicate what type of post-operative drainage was used, if any and for how many days the drainage was maintained. Multiple answers are possible.

- ☐ Ureteral splint
- ☐ TUC
- ☐ SPC
- ☐ Double J catheter
- ☐ Other: …..
- ☐ No postoperative drainage
- ☐ Unknown

Graft function

1.16 Data of discharge: Enter the date the patient was discharged from the hospital after the admission for the kidney transplantation. (DD-MM-YYYY)

1.17 eGFR at discharge: Enter the estimated glomerular filtration rate at the day of discharge (question 1.16) or if not available the day before discharge.
Use the Schwartz formula to calculate this eGFR.

1.18 Lowest eGFR: please enter the lowest eGFR between end of transplantation and discharge from the hospital.
Use the Schwartz formula to calculate this eGFR.

1.19 Date of last follow-up contact: Please enter the data the patient was last seen by a clinician for follow-up of the kidney transplantation (either nephrology, urology or pediatric surgery). (DD-MM-YYYY)
This might be an outpatient clinic visit or admission.

1.20 eGFR at last follow-up: Enter the most recent known eGFR of this patient.
Use the Schwartz formula to calculate this eGFR.
If the graft had been lost during follow-up, enter 0.

Immunosuppression

1.21 Please select the medication that was given as per-operative induction therapy

- ☐ Basiliximab
- ☐ Daclizumab
- ☐ ATG/ALG
- ☐ Alemtuzumab
- ☐ Other: ….
- ☐ No induction therapy

- ☐ Unknown

1.22 Please select the medication that was described at the moment the patient was discharged after the admission for the renal transplantation, multiple answers are possible.

- ☐ Tacrolimus
- ☐ Mycophenolate mofetil
- ☐ Prednisone
- ☐ Azathioprine
- ☐ Everolimus
- ☐ Cyclosporine
- ☐ Other: …..
- ☐ No immunosuppressants at discharge
- ☐ Unknown

<u>Infections</u>

1.23 CMV serological status donor prior to transplantation (IgG positive/negative)

- Positive
- Negative
- Unknown

1.24 EBV serological status donor prior to transplantation (IgG positive/negative)

- Positive
- Negative
- Unknown

1.25 CMV serological status recipient prior to transplantation (IgG positive/negative)

- Positive
- Negative
- Unknown

1.26 EBV serological status recipient prior to transplantation (IgG positive/negative)

- Positive
- Negative
- Unknown

1.27 EBV Infection during follow up. Enter If any EBV Infection (PCR proven) occurred during follow-up and whether this was de first time (primo Infection) or a reactivation.

- Yes, primo infection
- Yes, reactivation
- Yes, both primo infection and reactivation
- No
- Unknown

1.28 EBV prophylaxis. Enter whether the patient receive prophylactic medication for EBV at any moment during follow-up.

- Yes
- No
- Unknown

1.29 CMV Infection during follow up. Enter If any CMV Infection (PCR proven) occurred during follow-up and whether this was de first time (primo Infection) or a reactivation.

- Yes, primo infection
- Yes, reactivation
- Yes, both primo infection and reactivation
- No
- Unknown

1.30 CMV prophylaxis. Enter whether the patient receive prophylactic medication for EBV at any moment during follow-up.

- Yes
- No
- Unknown

1.31 BKV Infection during follow up. Enter If any BKV Infection (PCR proven) occurred during follow-up and whether this was de first time (primo Infection) or a reactivation.

- Yes, primo infection
- Yes, reactivation
- Yes, both primo infection and reactivation
- No
- Unknown

1.32 Culture proven UTI with fever. Please indicate whether a clinical relevant UTI: a culture proven urinary tract infection with fever, occurred during follow-up.

- Yes
- No
- Unknown

<u>Rejection</u>

1.33 Rejection: Indicate whether a pathologically confirmed rejection (both chronic or acute) of the kidney graft occurred during follow-up.

- Yes
- No
- Unknown

If yes, add the date of the rejection (DD-MM-YYYY)

2: SUPPLEMENTARY TABLES AND FIGURES

Supplementary Table 2. Independent determinants of EBV infection during follow up: multivariate binary logistic analysis**.** EBV: Epstein-Barr-virus

	β-coefficient	Exp(B)	p-value
**Unequal EBV status donor/recipient**	Reference	Reference	.011
**Both donor and recipient EBV negative**	-.384	.681	.490
**Both donor and recipient EBV positive**	-1.034	.356	.003
**Duration of follow-up**	.027	1.027	.000
**Discharge with Prednisone**	1.016	2.761	.002
**Constant**	-2.329	.097	.000

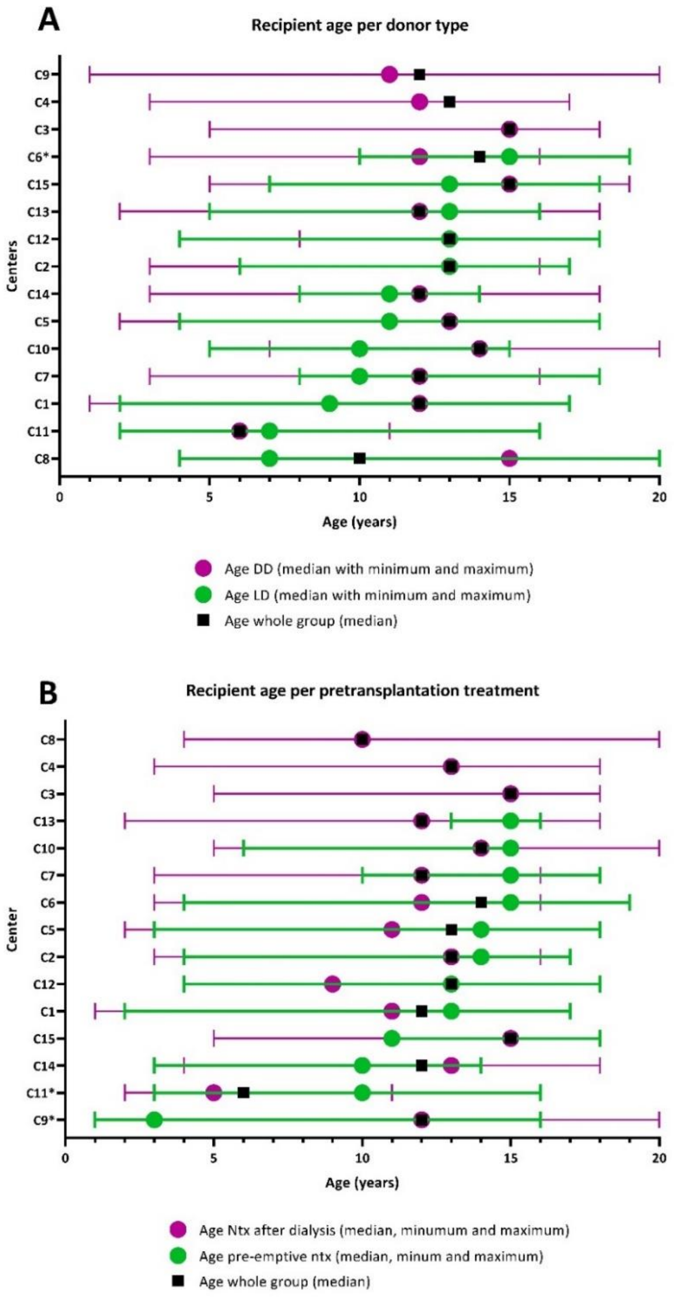

Supplementary Figure 1. Age per transplantation type.

A. Recipient age at transplantation per donor type. Median with minimum and maximum. * indicates significant age difference between LD and DD

B. Recipient age at transplantation per pre-transplantation treatment group. Median with minimum and maximum. * indicates significant age difference between pre-emptive transplantations and transplantation after dialysis

When n <3 no data were shown. DD: deceased donor, LD: living donor, Ntx: kidney transplantatio

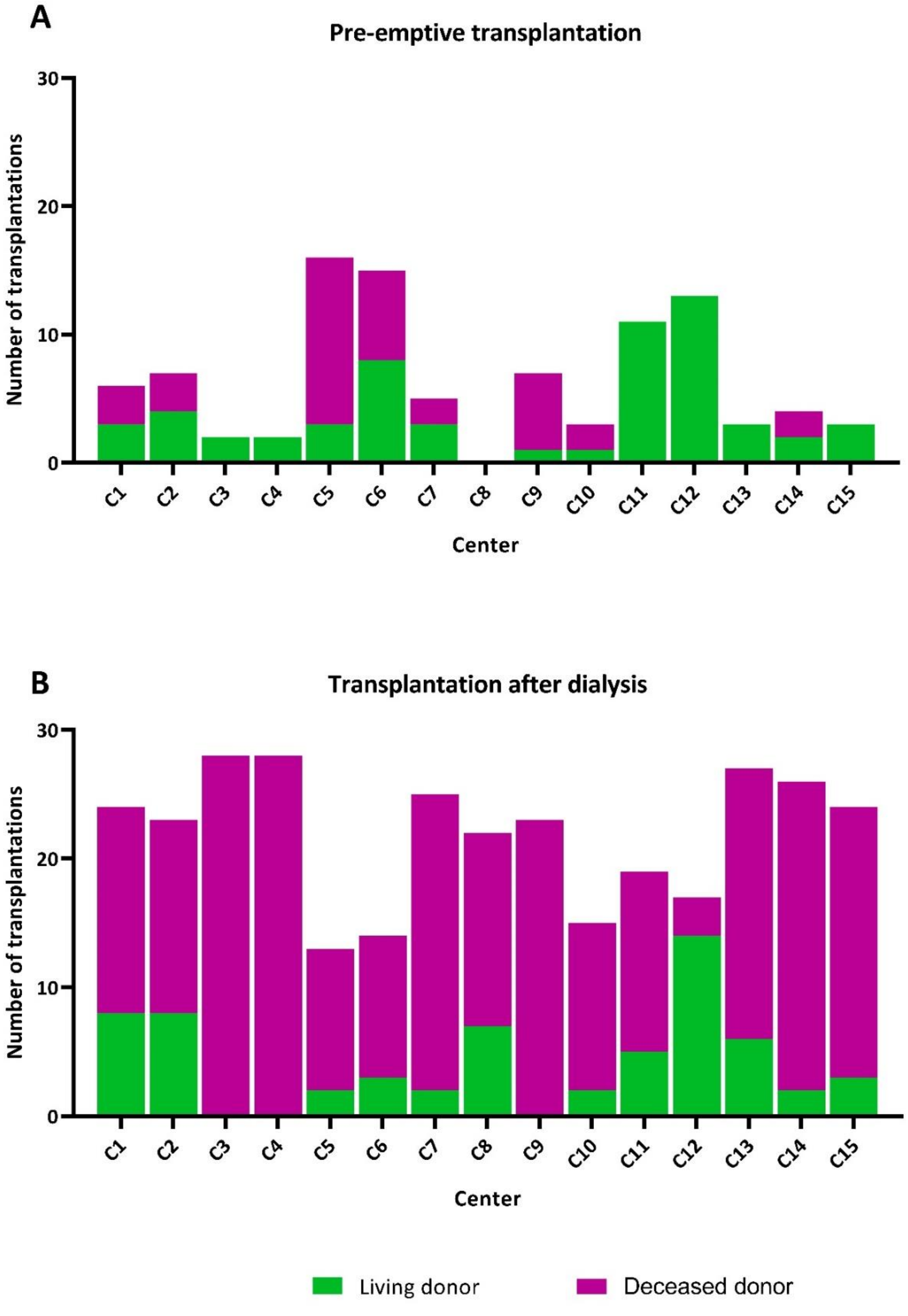

Supplementary Figure 2. Donor type per pre-transplantation treatment

A. Number of pre-emptive transplantations per center stratified by type of donor

B. Number of transplantations after dialysis per center stratified by type of donor

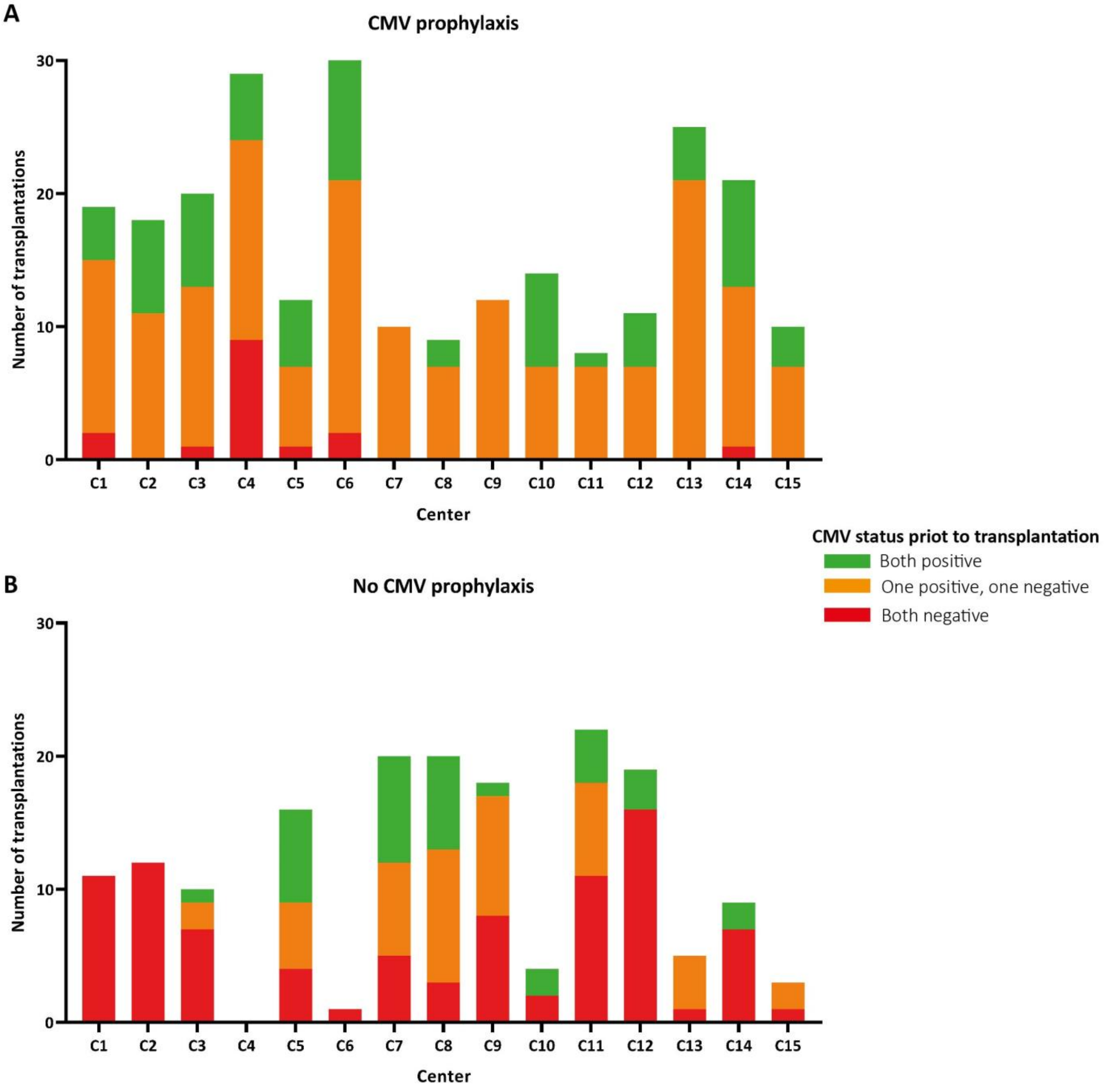

Supplementary Figure 3. Prescription of CMV prophylaxis per centre.

A. Number recipients that received CMV prophylaxis per centre stratified by the CMV status of donor and recipient prior to transplantation.

B. Number recipients that received not received CMV prophylaxis stratified by the CMV status of donor and recipient prior to transplantation.

CMV: cytomegalovirus

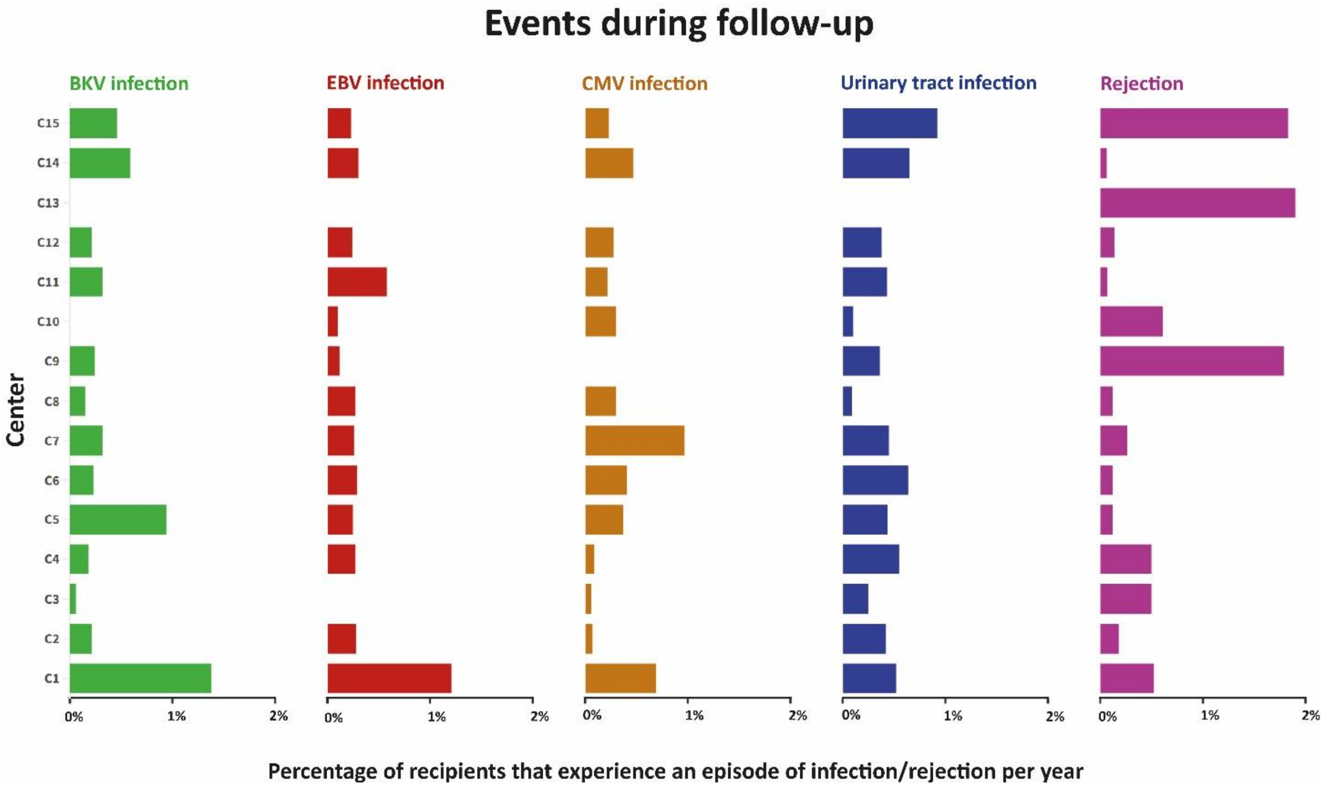

Supplementary Figure 4. Occurrence of events during follow-up (n=334). The percentage of recipients that experienced an episode of infection or rejection per year of follow-up per center. Patients with a follow-up <6 months were excluded from analysis. BKV: BK virus, EBV: Epstein-Barr virus, CMV: cytomegalovirus

3: STROBE STATEMENT

Checklist of items that should be included in reports of cross-sectional studies

	Item No	Recommendation	Page No
**Title and abstract**	1	(*a*) Indicate the study's design with a commonly used term in the title or the abstract	
		(*b*) Provide in the abstract an informative and balanced summary of what was done and what was found	
		**Introduction**	
Background/rationale	2	Explain the scientific background and rationale for the investigation being reported	
Objectives	3	State specific objectives, including any prespecified hypotheses	
		**Methods**	
Study design	4	Present key elements of study design early in the paper	
Setting	5	Describe the setting, locations, and relevant dates, including periods of recruitment, exposure, follow-up, and data collection	
Participants	6	(*a*) Give the eligibility criteria, and the sources and methods of selection of participants	
Variables	7	Clearly define all outcomes, exposures, predictors, potential confounders, and effect modifiers. Give diagnostic criteria, if applicable	
Data sources/ measurement	8*	For each variable of interest, give sources of data and details of methods of assessment (measurement). Describe comparability of assessment methods if there is more than one group	
Bias	9	Describe any efforts to address potential sources of bias	
Study size	10	Explain how the study size was arrived at	
Quantitative variables	11	Explain how quantitative variables were handled in the analyses. If applicable, describe which groupings were chosen and why	
Statistical methods	12	(*a*) Describe all statistical methods, including those used to control for confounding	
		(*b*) Describe any methods used to examine subgroups and interactions	
		(*c*) Explain how missing data were addressed	
		(*d*) If applicable, describe analytical methods taking account of sampling strategy	NA

		(*e*) Describe any sensitivity analyses	NA
**Results**			
Participants	13*	(a) Report numbers of individuals at each stage of study—eg numbers potentially eligible, examined for eligibility, confirmed eligible, included in the study, completing follow-up, and analysed	
		(b) Give reasons for non-participation at each stage	
		(c) Consider use of a flow diagram	NA
Descriptive data	14*	(a) Give characteristics of study participants (eg demographic, clinical, social) and information on exposures and potential confounders	Incorporated in all graphs and Table 1
		(b) Indicate number of participants with missing data for each variable of interest	In all legendas
Outcome data	15*	Report numbers of outcome events or summary measures	
Main results	16	(*a*) Give unadjusted estimates and, if applicable, confounder-adjusted estimates and their precision (eg, 95% confidence interval). Make clear which confounders were adjusted for and why they were included	Table 1, all figures
		(*b*) Report category boundaries when continuous variables were categorized	NA
		(*c*) If relevant, consider translating estimates of relative risk into absolute risk for a meaningful time period	NA
Other analyses	17	Report other analyses done—eg analyses of subgroups and interactions, and sensitivity analyses	
**Discussion**			
Key results	18	Summarise key results with reference to study objectives	
Limitations	19	Discuss limitations of the study, taking into account sources of potential bias or imprecision. Discuss both direction and magnitude of any potential bias	
Interpretation	20	Give a cautious overall interpretation of results considering objectives, limitations, multiplicity of analyses, results from similar studies, and other relevant evidence	
Generalisability	21	Discuss the generalisability (external validity) of the study results	
**Other information**			
Funding	22	Give the source of funding and the role of the funders for the present study and, if applicable, for the original study on which the present article is based	NA
